# The Efficacy and Safety of Pharmacological Treatments for Restless Legs Syndrome: Systemic Review and Network Meta-Analysis

**DOI:** 10.3389/fnins.2021.751643

**Published:** 2021-10-26

**Authors:** Xuan Zhou, Juncong Du, Yi Liang, Chengcheng Dai, Lili Zhao, Xi Liu, Changhong Tan, Lijuan Mo, Lifen Chen

**Affiliations:** Department of Neurology, The Second Affiliated Hospital of Chongqing Medical University, Chongqing, China

**Keywords:** restless legs syndrome, pharmacological interventions, efficacy, international RLS study group rating scale, network meta-analysis, safety

## Abstract

Although various drugs are currently used for restless legs syndrome (RLS) in clinic, selecting appropriate drugs for patients is difficult. This network meta-analysis (NMA) aimed to compare the efficacy and safety of different drugs. After literature searching and screening, 46 trials, including 10,674 participants are included in this NMA. The pooled results showed that, compared with placebo, only levodopa is inefficient to relieve symptoms of RLS. Cabergoline decreases IRLS scores to the greatest extent among all drugs (MD −11.98, 95% CI −16.19 to −7.78). Additionally, pramipexole is superior to ropinirole in alleviating symptoms of RLS (MD −2.52, 95% CI −4.69 to −0.35). Moreover, iron supplement alleviates RLS symptoms significantly compared with placebo in patient with iron deficiency (MD −5.15, 95% CI −8.99 to −1.31), but not for RLS patients with normal serum ferritin level (MD −2.22, 95% CI −6.99 to 2.56). For primary RLS, these drugs are also effective, while there is insufficient data to analyze drug efficacy in secondary RLS. We analyzed risk of common adverse effects of drugs including nausea, somnolence, fatigue, headache and nasopharyngitis. Alpha-2-delta ligands and DAs are favorable choices for both primary and secondary RLS because of their significant efficacy and good tolerability. Iron supplement can significantly alleviate symptoms of RLS patients with iron deficiency than placebo. We recommend gabapentin, gabapentin enacarbil, and pregabalin for clinicians for first consideration mainly because that they rarely cause augmentation. Oxycodone-naloxone could be considered in patients with severe or very severe RLS who failed in treatment with above drugs.

## Introduction

Restless legs syndrome (RLS), known as a neurological sensorimotor disorder, is characterized by an urgency to move the limbs with a sensation of discomfort, occurring more frequently during rest, in the evening or at night, mostly involving the lower limbs. In severe cases, it may also extend to the daytime and affect the upper limbs (American Academy of Sleep Medicine, 2014). The estimated incidence of RLS diagnosed as a set of symptoms meeting the minimal diagnostic criteria of the International RLS Study Group ranged from 3.9 to 14.3% (Ohayon et al., 2012) and it was reported to be nearly 7.2% in European and American adult populations (Allen et al., 2005). There are two forms of RLS, primary and secondary. Primary restless legs syndrome occurs in adults and possibly with family history, while secondary RLS occurs in patients mainly suffered from polyneuropathy, neurodegenerative disease, chronic kidney disease, iron deficiency, pregnancy and drugs, such as antipsychotics and antidepressants (Oka and Loue, 2009; Trenkwalder et al., 2016). The RLS not only affects sleep quality, but also induces mood disorders, affects quality of life, and increases financial burden.

There are various pharmacological therapeutic options for RLS. Levodopa, a most commonly used dopaminergic agent, was once the only drug approved for RLS in some European countries (García-Borreguero et al., 2012a,b). However, dopamine agonists (DAs), (Trenkwalder et al., 2005; Vignatelli et al., 2006) have replaced levodopa and become the first-line treatment in recent years. In addition, gabapentin is now proven effective for primary or secondary RLS (Razazian et al., 2015). With abundant treatment options existing, selecting appropriate treatment for patients is difficult. Iftikhar et al. ever conducted a network meta-analysis to compare the efficacy of dopaminergic drugs and alpha-2-delta ligands (α-2-δ ligands) for alleviating RLS (Iftikhar et al., 2017). They found pramipexole, ropinirole, rotigotine, gabapentin enacarbil and pregabalin effective for alleviating RLS, but there was no significant difference between these drugs. However, they did not compare other commonly-used RLS treatments, such as iron supplements, gabapentin and levodopa, which are also considered as effective treatments for primary or secondary RLS (Earley et al., 2000; García-Borreguero et al., 2012a,b; Razazian et al., 2015). Notably, iron supplement has been proven effective for RLS patients with decreased serum level of ferritin, but its effectiveness in RLS patients with normal serum ferritin level is unclear (Earley et al., 2000). Moreover, all the currently available drugs for treatment of RLS presented various adverse effects, including augmentation, dizziness, nausea, headache, fatigue, and somnolence (Trenkwalder et al., 2018; Winkelmann et al., 2018). These adverse effects should also be taken into consideration of selection of treatments for RLS patients.

Network meta-analysis (NMA) is a relatively recent quantitative methodology, which allows for comparisons of multiple interventions in a single analysis compared with pair-wise and direct comparisons of traditional meta-analysis, based on the assumption of transitivity. For example, if study about comparison of A and B is absent, while comparison of A and C as well as comparison of B and C are existed, then A and B can be compared indirectly (Li et al., 2011). NMA provides a global estimate of several comparative interventions through combing direct and indirect comparison together, making it an increasingly popular method in comparative inventions and possibly providing the researcher the best choice among all the interventions (Caldwell et al., 2005; Li et al., 2011). Therefore, we performed this NMA to compare the efficacy and safety of different pharmacological treatments more comprehensively, which may assist clinicians in treatment of RLS patients.

## Materials and Methods

### Search Strategy

This NMA is performed following the guidelines of the Preferred Reporting Items for Systematic Reviews and Meta-analyses (Hutton et al., 2015).

Relevant articles publicated in English or Chinese, from January, 2000 to December, 2020 were systematically searched in PubMed, EMBASE and Chinese National Knowledge Infrastructure. The search strategy was designed as follows: [(restless legs syndrome) OR (Willis-Ekbom disease)] AND (treatment OR medication OR therapy). Reference lists of the relevant articles were also hand-searched for potentially related articles.

### Study Selection

Inclusion criteria were as follows: (1) Randomized control trials (RCTs); (2) Trials conducted comparison between drugs, or between drugs and placebo; (3) With at least 5 participants in each group; (4) All participants are 18-year-old or older; (5) The efficacy of treatments was assessed using the International RLS Study Group Rating Scale (IRLS); (6) Mean difference (MD) and standard deviation (SD) should be provided or could be calculated; (7) In studies provided standard error (SE) instead of SD, SD was calculated as follows: $SD=SE\times \sqrt{}number\;of\;participants$; (8) In studies provided only 95% confidence interval (95% CI), SD was calculated as follows: *SD* = (*the upper limit of* 95*% CI* − *MD*)/1.96.

The exclusion criteria were as follows: (1) Cross-sectional studies, cohort studies, case reports, conferences, reviews or letters/commentary; (2) Articles did not report MD or SD of IRLS, or MD and SD could not be calculated based on provided information.

Two researchers (XZ, LM) independently screened titles and abstracts to exclude the irrelevant studies. Then they viewed the full texts of the remaining candidate studies for eligible investigations. Discordance was solved by consensus with the help of the third reviewer (LC).

### Data Extraction and Quality Assessment

The following information was extracted from each study: name of first author, publication year, country, diagnosis, drugs, sample size, sex proportion, mean age of participants, study duration, MD and SD of IRLS, and number of individuals who experienced adverse effects and the type of adverse effects (Supplementary Table 1).

For studies reported multiple dosage of a drug, the dosage with most favorable outcome was included. The Cochrane Collaborations tool for RCTs was used to evaluate the quality of the included studies by two reviewers mentioned above independently (The Cochrane Collaboration, 2011) (http://www.Cochrane-handbook.org). The domain-based evaluation included the following six criteria: selection bias (included sequence generation and allocation concealment), performance bias, detection bias, attrition bias, reporting bias and other bias. Each domain was graded as “low risk,” “high risk,” or “unclear.” Review Manager 5.4 software (The Cochrane Collaboration, Oxford, UK) was used to run the Cochrane Collaborations tool. Discordance was solved by consensus with the help of the third reviewer.

### Statistical Analysis

We performed a NMA in a frequentist framework with Stata15 SE version software (Stata Corporation, College Station, USA) using the network meta command and Stata routines available at http://www.mtm.uoi.gr (Shim et al., 2017) with parameter estimation using restricted maximum likelihood (White, 2015). Frequentist NMA summarize direct and indirect evidence together using inverse variance method, namely, inverse variance pf each study is used as weight to calculate the weighted average of each study effect, variance of the overall effect equals to reciprocal of sum of weight. A random-effect model was used to combine both direct (within-trial) and indirect (across-trial) evidence on efficacy of different drugs (Salanti, 2012). The pooled estimation of the effect size for NMA was presented in MD together with 95% CI for continuous outcomes, which were used to assess the efficacy of different treatments. Odds ratio (OR) together with 95% CI were used to assess the risk of adverse effects. Results with 95% CI did not across the null value was considered statistically significant. Consistency between direct and indirect evidence is a crucial assumption in NMA, which means, for closed loop in the NMA, evidence from direct and indirect comparisons should agree on average (White et al., 2012). The consistency of direct and indirect evidence for efficacy of drugs in this NMA was explored by node-splitting method for closed loop. *P* < 0.05 or inconsistency factor is away from zero suggested significant inconsistency, and further investigation would be necessary (Dias et al., 2010). Surface under the cumulative ranking (SUCRA) is able to estimate the cumulative ranking for each treatment by a simple graphical display and numerical summary. The value of SUCRA ranged from 0 to 1, with one indicating that a treatment is certain to be rank the first and zero that a treatment is certain to rank the last (Salanti et al., 2011). We also evaluated the relative ranking of different drugs for the treatment of RLS based on SUCRA. We assessed potential small-trial effects and publication bias by using comparison-adjusted funnel plots for network meta-analysis.

## Results

### Study Selection

**Figure 1:**
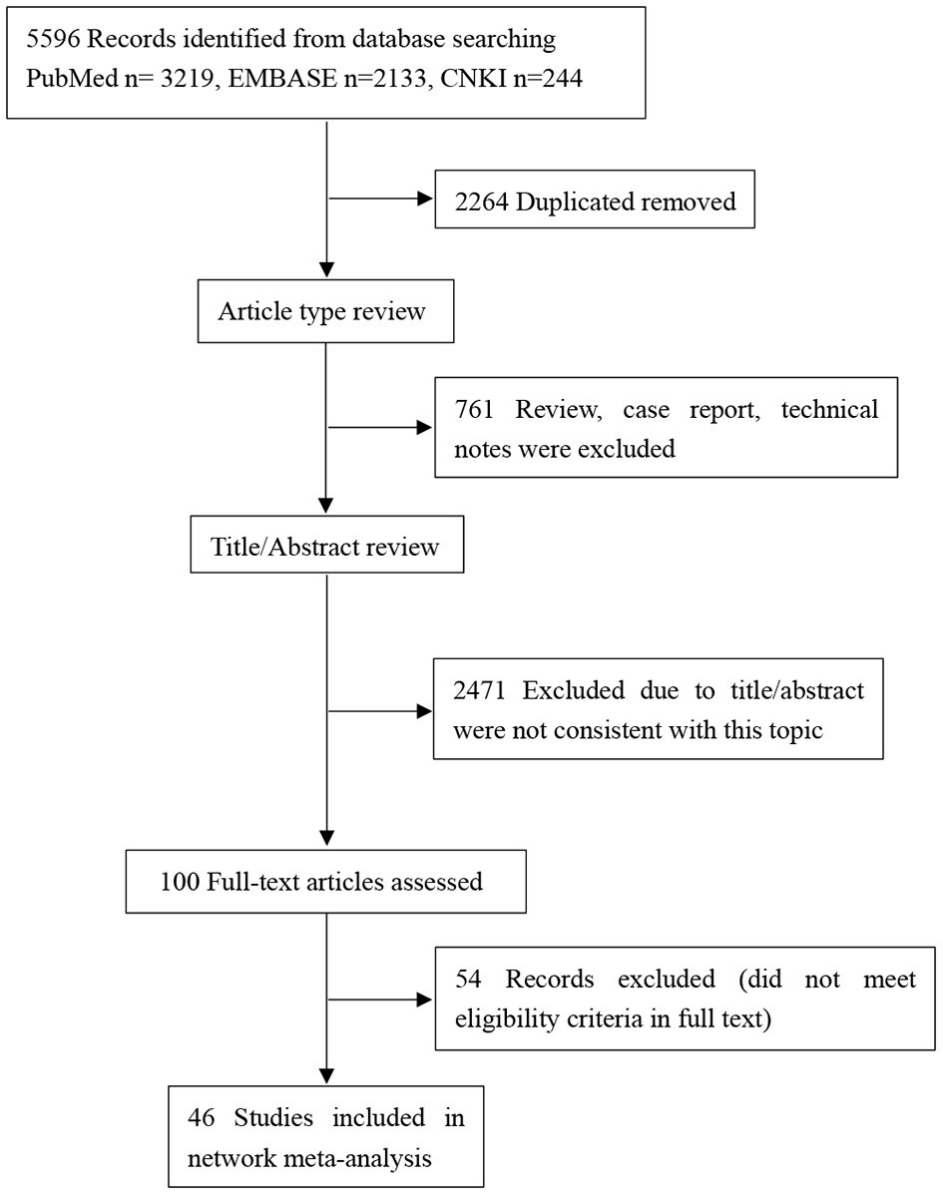
PRISMA flow-chart showing selection of articles for network meta-analysis.

The initial literature search identified 5,596 potentially relevant articles (3,219 from PubMed, 2,133 from Embase, 244 from CNKI. After removing 2,264 duplicates, the titles and abstracts of the remaining 3,332 articles were primarily screened. 100 potentially eligible articles were further estimated by full text view. Finally, 46 RCTs including a total of 10,674 participants were included (Figure 1).

### Study Characteristics

The included studies investigated 10 different drugs, including gabapentin, gabapentin enacarbil, ropinirole, rotigotine, pramipexole, iron, levodopa, pregabalin, cabergoline and oxycodone-naloxone. All the included studies were published during 2004 to 2018. The sample sizes of the included studies ranged from 18 to 752. One of the included studies had three arms, while the rest studies all had two arms. 34 studies were conducted in European countries or USA, eight studies were conducted in Asia, four studies did not report the region where they were conducted (Supplementary Table 1). Among the included studies, 42 studies (91.3%) were placebo-controlled trials, the remaining four studies compared different drugs. Among the included studies, 37 studies were based on primary RLS patients, two studies were based on secondary RLS patients (patients with chronic kidney disease on hemodialysis), seven studies did not report the specific type of RLS.

The quality evaluating of the included studies were shown in Supplementary Figure 1. None of the included studies was found to have high risk of bias among all the 46 included trails, with four studies were identified as moderate risk of bias. Meanwhile, the rest of 42 studies were considered at low risk of bias.

### Network Meta-Analysis for Efficacy of Different Drugs

**Figure 2:**
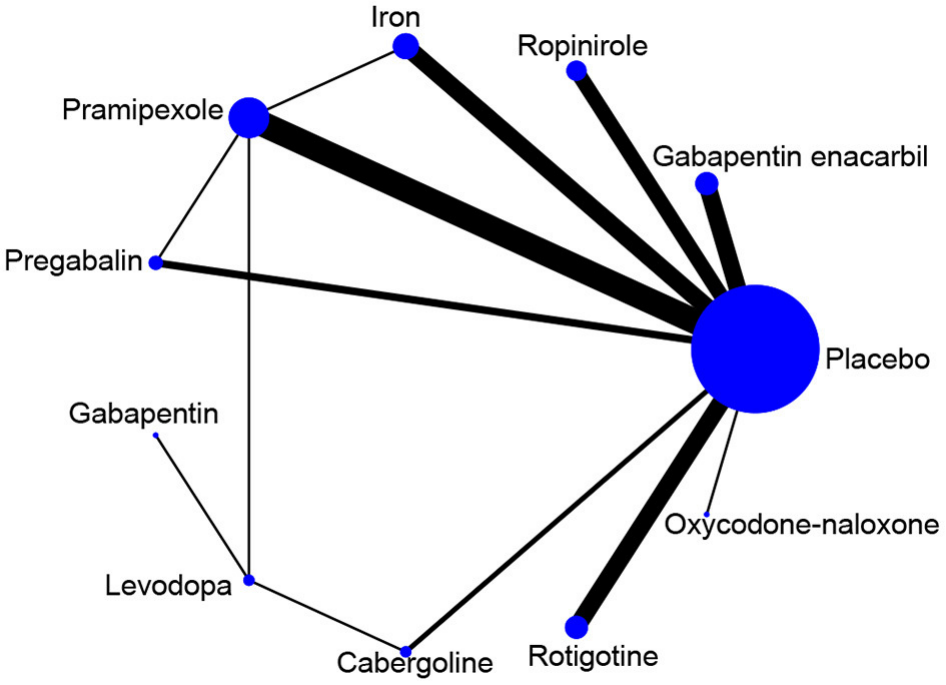
Network map of available comparisons. Width of lines is proportional to the number of trials including every pair of treatments (direct comparisons). Size of every circle size is proportional to the total number of randomly assigned participants (i.e., sample size).

**Figure 3:**
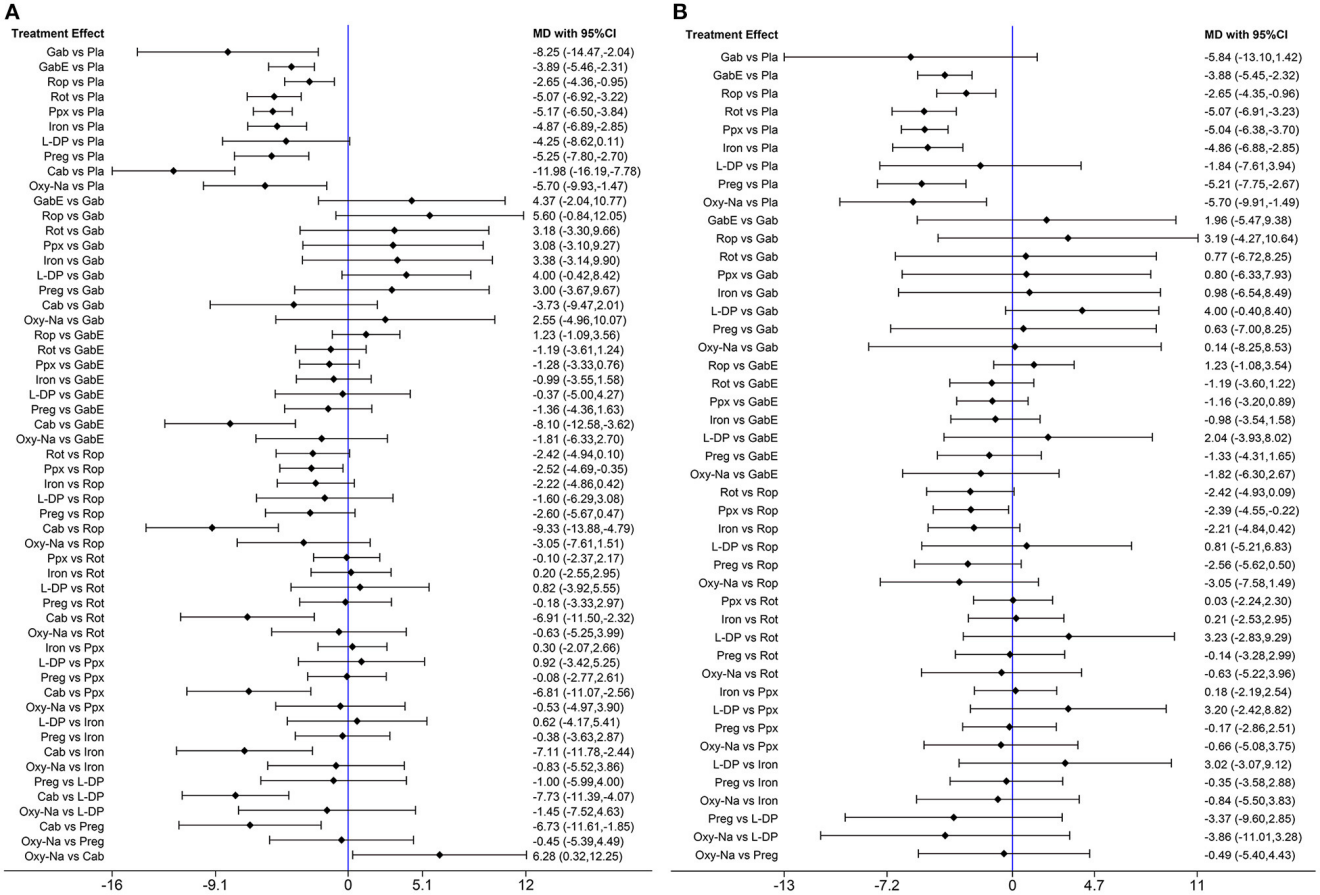
**(A)** Forest plots of pairwise meta-analysis for all included drugs. **(B)** Forest plots of pairwise meta-analysis for the remaining drugs after excluding cabergoline. (Gab, Gabapentin; GabE, Gabapentin enacarbil; L-DP, Levodopa; Rot, Rotigotine; Ppx, Pramipexole; Cab, Cabergoline; Rop, Ropinirole; Preg, Pregabalin; Oxy-Na, Oxycodone-naloxone; Pla, Placebo).

The network diagram of all the included drugs is presented in Figure 2. There are 11 nodes and 13 direct treatment comparisons in the network plot. Each node represents a drug, and the line between nodes reflects direct comparison. The size of nodes and width of lines are proportional to participant numbers and trial numbers, respectively. The results of NMA indicate that, compared with placebo, only levodopa is inefficient to relieve symptoms of RLS, while the others significantly decrease the scores of IRLS. Cabergoline decreases IRLS scores to the greatest extent among all drugs (MD −11.98, 95% CI −16.19 to −7.78), which shows significantly better effect than the other drugs. Additionally, pramipexole is superior to ropinirole in alleviating symptoms of RLS (MD −2.52, 95% CI −4.69 to −0.35) (Figure 3A).

**Figure 4:**
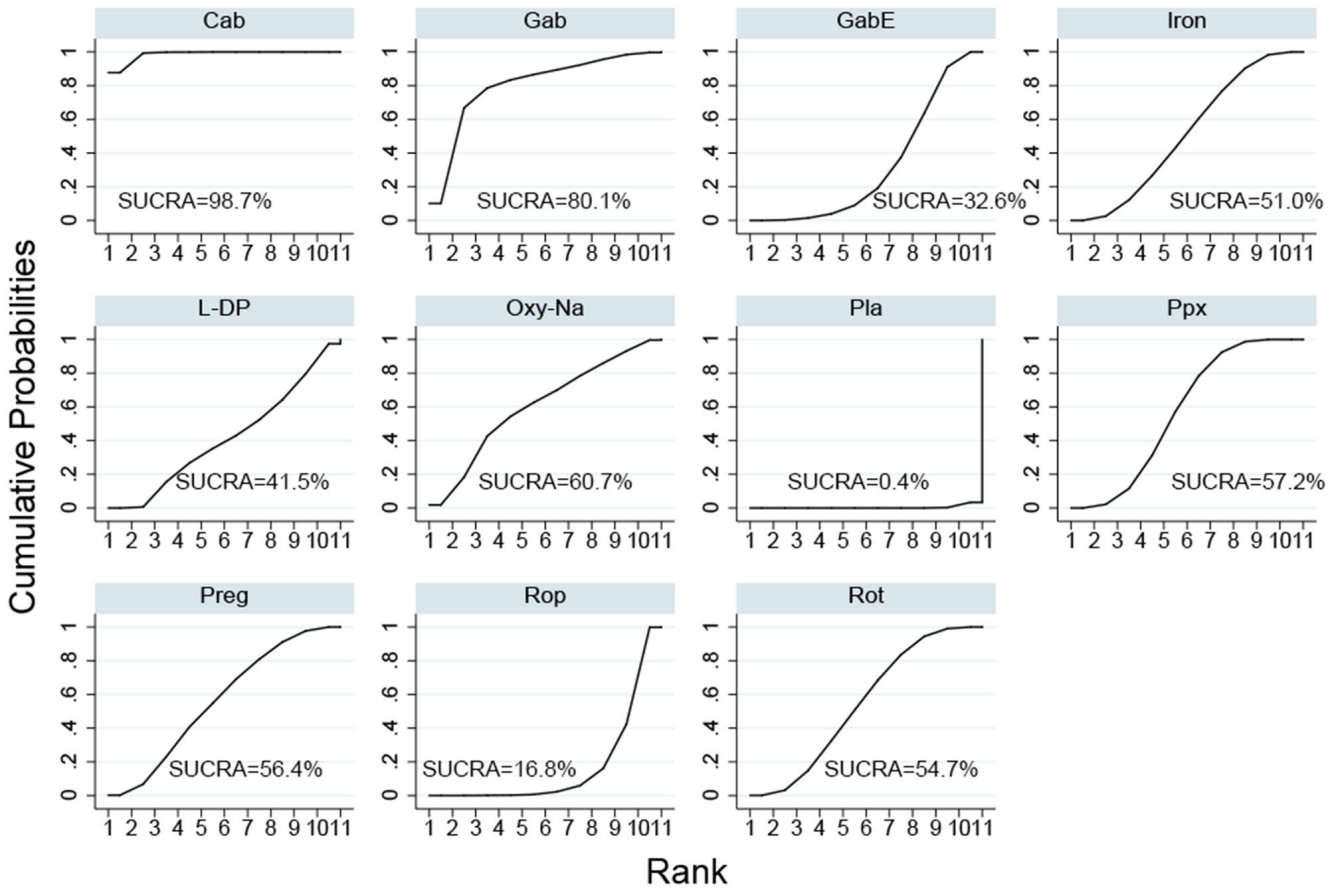
Surface under the cumulative ranking curve (SUCRA) of all included drugs. (Gab, Gabapentin; GabE, Gabapentin enacarbil; L-DP, Levodopa; Rot, Rotigotine; Ppx, Pramipexole; Cab, Cabergoline; Rop, Ropinirole; Preg, Pregabalin; Oxy-Na, Oxycodone-naloxone; Pla, Placebo).

The SUCRA ranks of the efficacy of all the investigated drugs is shown in Figure 4 and Supplementary Table 3, cabergoline ranks first, sequentially followed by gabapentin, oxycodone-naloxone, pregabalin, pramipexole, rotigotine, iron supplement, levodopa, gabapentin enacarbil, and ropinirole.

**Figure 5:**
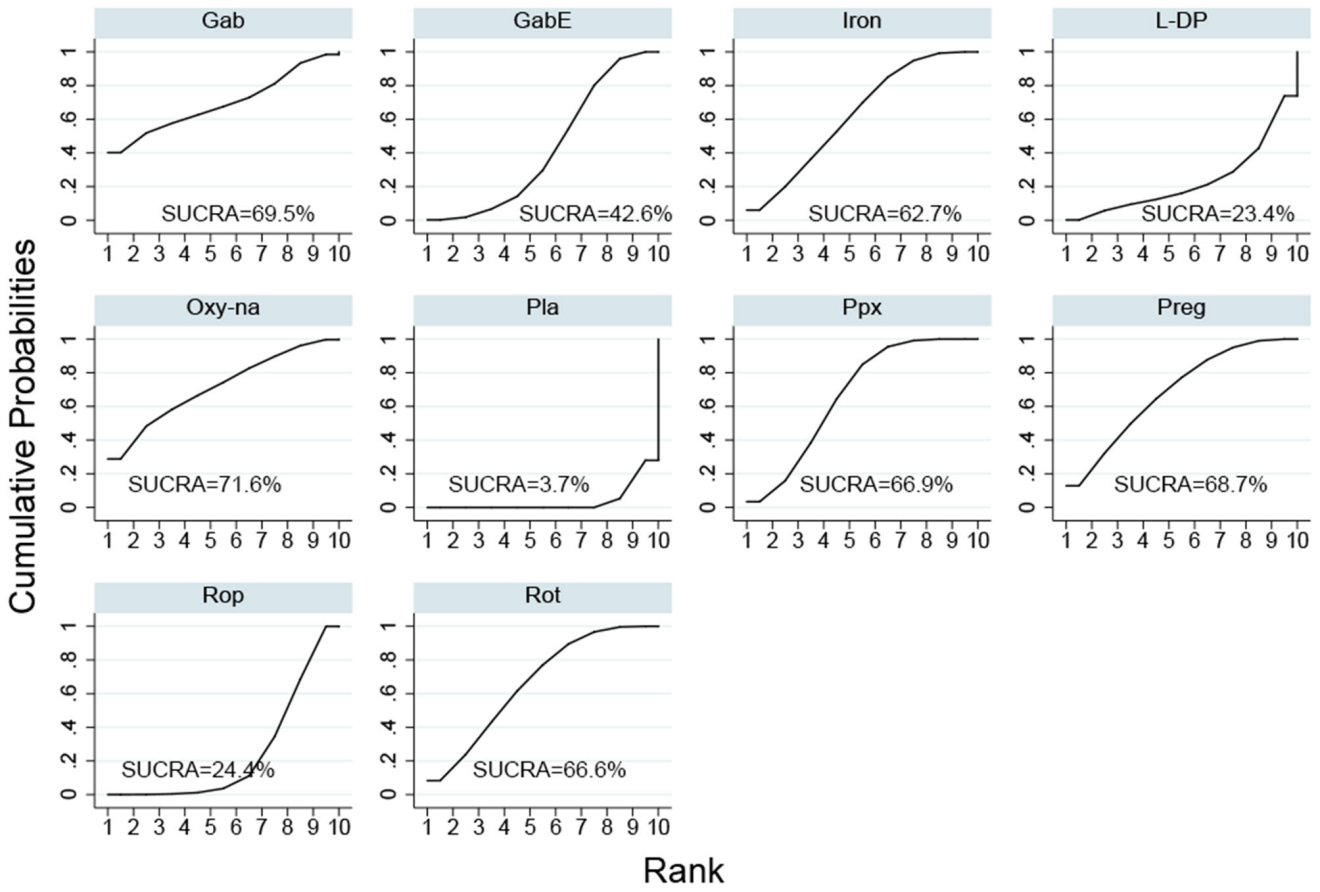
Surface under the cumulative ranking curve (SUCRA) of the rest of drugs after excluding cabergoline. (Gab, Gabapentin; GabE, Gabapentin enacarbil; L-DP, Levodopa; Rot, Rotigotine; Ppx, Pramipexole; Cab, Cabergoline; Rop, Ropinirole; Preg, Pregabalin; Oxy-Na, Oxycodone-naloxone; Pla, Placebo).

Cabergoline is not widely approved for treating RLS due to its potential side effects (Winkelmann et al., 2018). Therefore, we performed a NMA excluding cabergoline. The pooled results show that all drugs, except, gabapentin and levodopa, are effective in alleviating symptoms of RLS compared with placebo (Figure 3B). The SUCRA suggests that oxycodone-naloxone is optimal in alleviating symptoms of RLS, followed by gabapentin, pregabalin, pramipexole, rotigotine, iron supplement, gabapentin enacarbil, ropinirole and levodopa (Figure 5; Supplementary Table 4).

**Figure 6:**
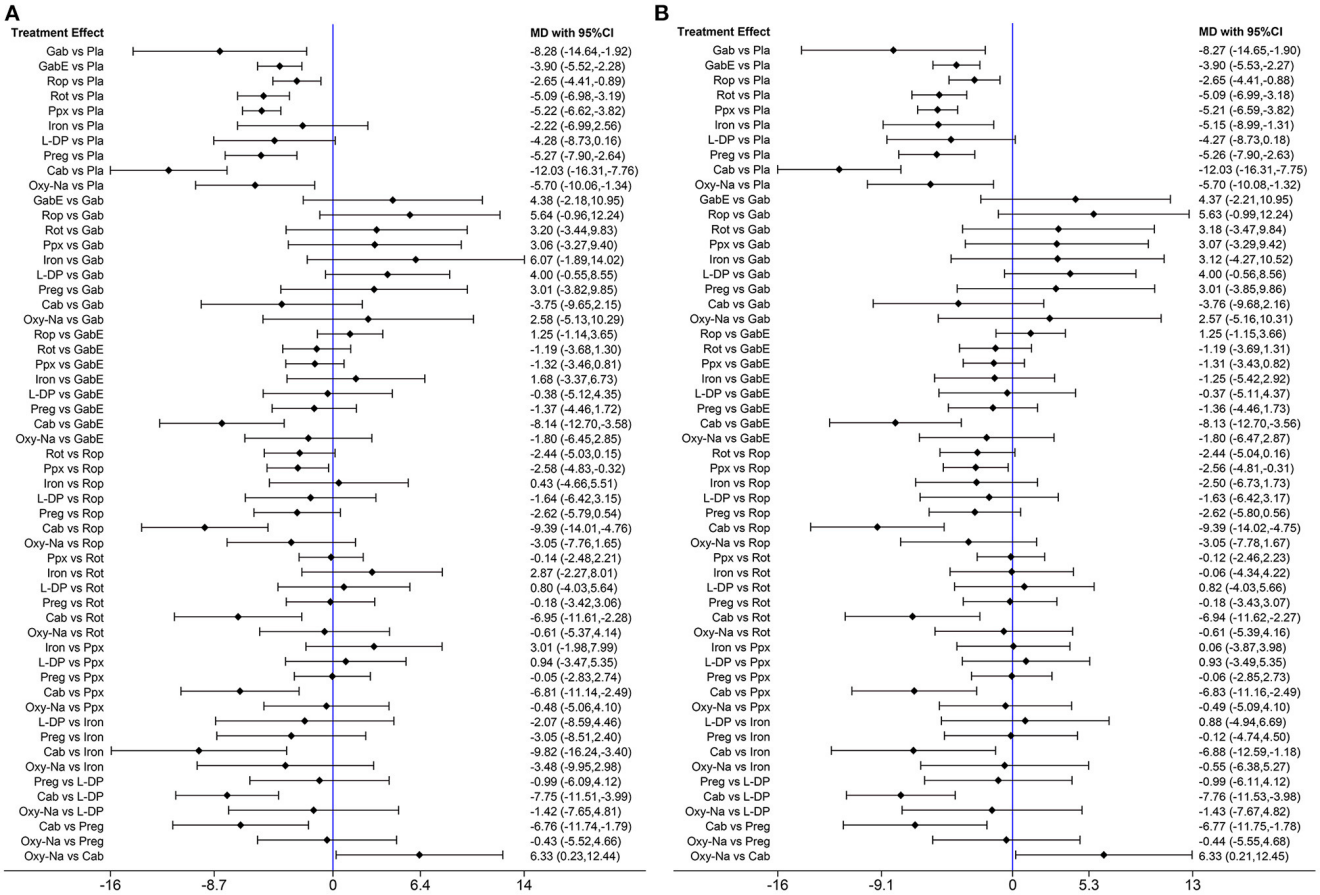
**(A)** The network analysis result after removing studies with uncertain serum ferritin level or low serum ferritin level (studies iron supplement for RLS with serum ferritin level upper than 45 ug/l included). **(B)** The network analysis result of different drugs in treatment of RLS patients with low serum ferritin level. (Gab, Gabapentin; GabE, Gabapentin enacarbil; L-DP, Levodopa; Rot, Rotigotine; Ppx, Pramipexole; Cab, Cabergoline; Rop, Ropinirole; Preg, Pregabalin; Oxy-Na, Oxycodone-naloxone; Pla, Placebo).

Additionally, we analyzed whether iron supplement is effective to relieve symptoms in RLS patients with normal serum ferritin level. However, the result showed that iron supplement could not alleviate RLS symptoms significantly compared with placebo (MD −2.22, 95% CI −6.99 to 2.56) (Figure 6A). We also analyzed the effect of iron supplement on RLS with lower serum ferritin level (<45 ug/L), the result showed iron supplement can alleviate symptoms of RLS patients significantly compared with placebo (MD −5.15, 95% CI −8.99 to −1.31) (Figure 6B).

**Figure 7:**
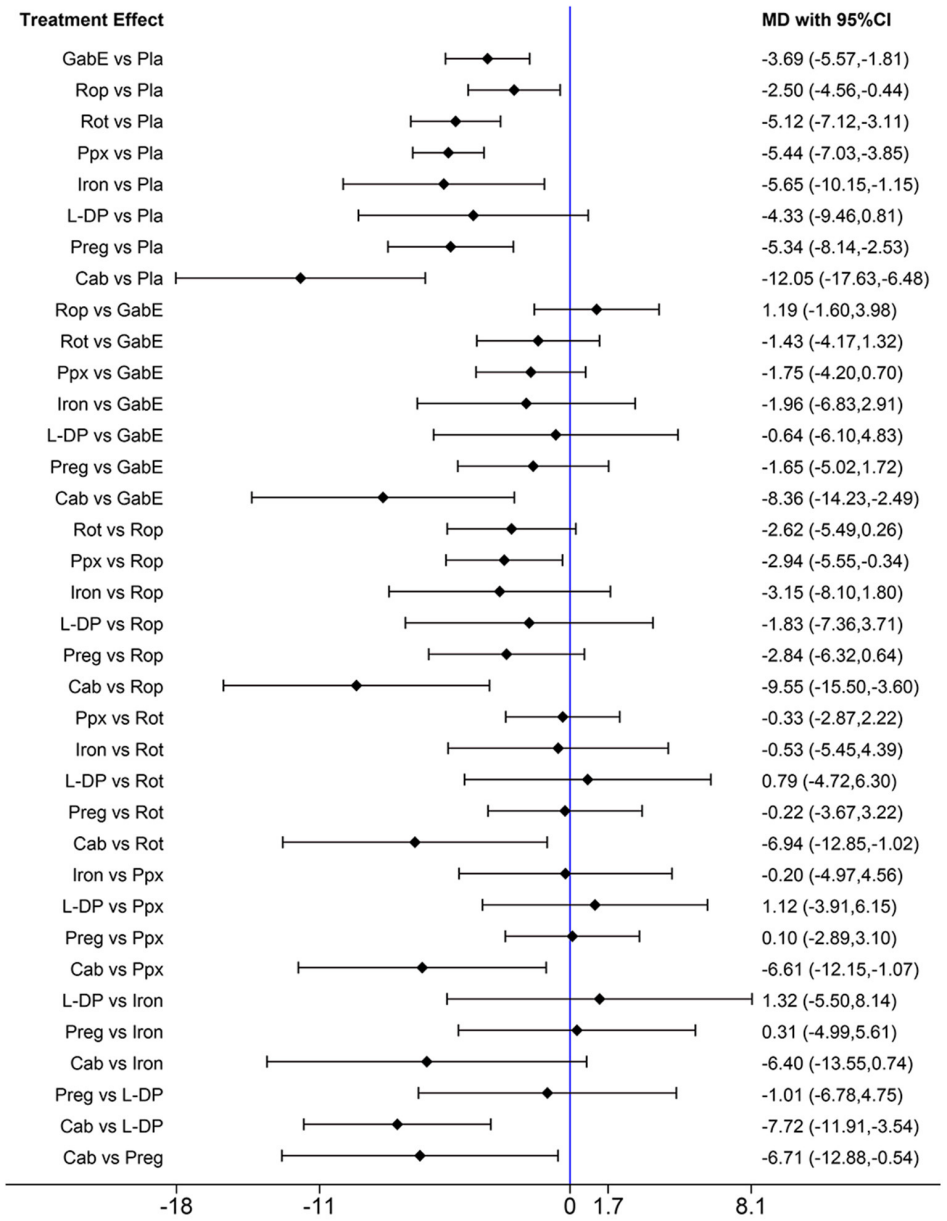
Forest plots of pairwise meta-analysis for different drugs in treatment of primary RLS patients. (Gab, Gabapentin; GabE, Gabapentin enacarbil; L-DP, Levodopa; Rot, Rotigotine; Ppx, Pramipexole; Cab, Cabergoline; Rop, Ropinirole; Preg, Pregabalin; Oxy-Na, Oxycodone-naloxone; Pla, Placebo).

To clarify the effect of the drugs on primary or secondary RLS, we performed further analysis. When only studies on primary RLS are included, the pooled results indicate that cabergoline, gabapentin enacarbil, rotigotine, ropinirole, pramipexole, iron and pregabalin are still effective to alleviate symptoms of RLS (Figure 7). SUCRA ranks cabergoline (98.9%) as the best drug for alleviating symptoms of primary RLS, and ranks pramipexole (64.7%) at the second place, followed by iron supplement (63.1%) (Supplementary Figure 2). Gabapentin is not analyzed due to lack of data. There is insufficient data to analyze effect of drugs on secondary RLS.

**Figure 8:**
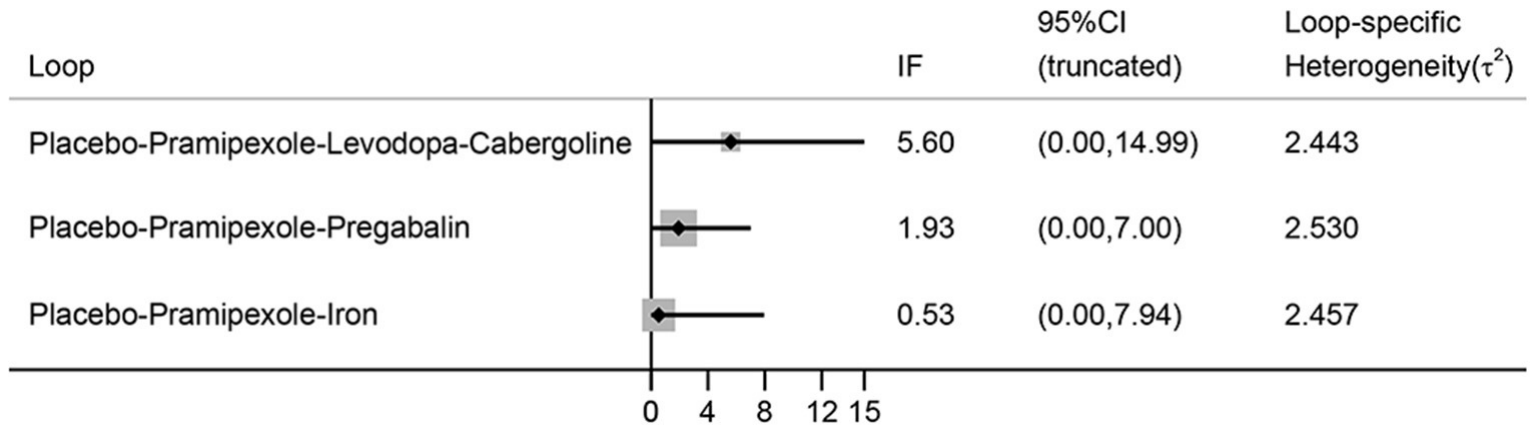
The inconsistency plot for direct and indirect comparisons. (IF, inconsistent factor; CI, confidence interval).

No evidence of inconsistency is indicated in the NMA, because all the 95% CIs of inconsistent factor include zero and *P* > 0.05 (Figure 8).

**Figure 9:**
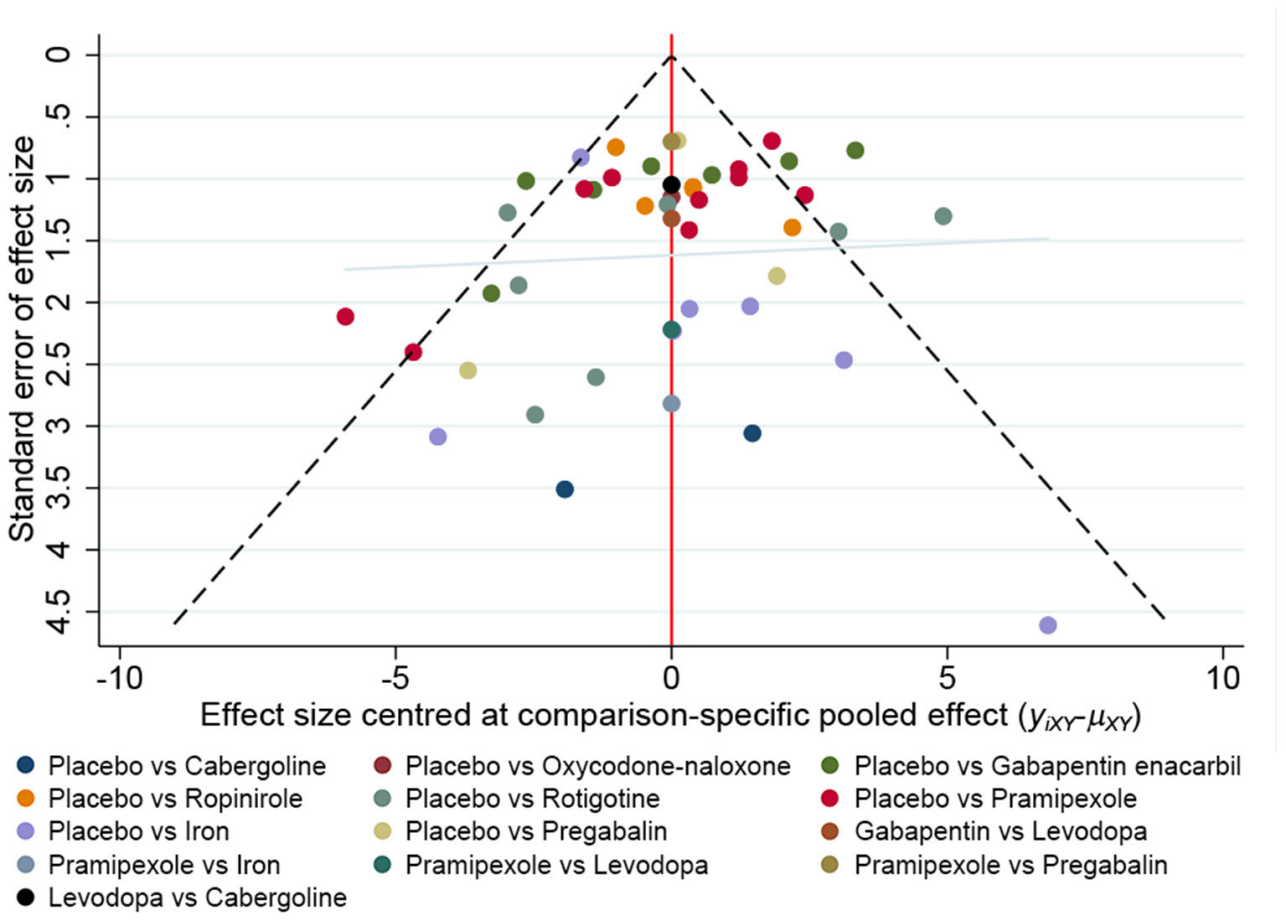
A funnel plot to confirm the risk of publications bias for included literatures.

There is no obvious asymmetry in the funnel graph, indicate less likelihood of publication bias (Figure 9).

### Network Meta-Analysis for Safety of Drugs

Due to limited data, we only analyzed the common adverse effects of drugs, including nausea, fatigue, headache, dizziness, somnolence and nasopharyngitis. As shown in Supplementary Table 2, in patients receiving non-ergot derived DAs, incidence of nausea is increased (rotigotine: OR 2.50, 95% CI 1.28–4.87; pramipexole: OR 3.19, 95% CI 1.53–6.67; ropinirole: OR 5.02, 95% CI 2.50–10.06). While patients receiving pregabalin and gabapentin enacarbil show significantly increased incidence of somnolence (OR 4.67, 95% CI 1.23–17.66; OR 3.56, 95% CI 1.77–7.14, respectively). In patients treated by ropinirole and oxycodone-naloxone, incidence of fatigue is increased (OR 2.74, 95% CI 1.56–4.81; OR 2.78, 95% CI 1.52–5.08, respectively). Patients receiving gabapentin enacarbil and ropinirole presented increased incidence of dizziness (OR 3.70, 95% CI 2.08–6.58; OR 2.53, 95% CI 1.05–6.10, respectively). No drug shows increased risk of headache and nasopharyngitis.

## Discussion

Based on 46 RCTs, our NMA explore the efficacy of current pharmacological treatments measured by IRLS in patients with RLS, and find that α-2-δ ligands, DAs, oxycodone-naloxone and iron supplement are effective to relieve the symptoms of RLS compared with placebo. Among the analyzed 10 drugs, cabergoline showed the greatest efficacy in relieving symptoms of RLS. However, due to its adverse effect, we also performed another analysis without cabergoline, which found that oxycondone-naloxone and gabapentin presented favorable efficacy in relieving symptoms of RLS. Levodopa is proven lacking in efficacy for RLS compared with placebo. Furthermore, iron supplement only alleviates symptoms of RLS patients with iron deficiency (serum ferritin levels lower than 45 ug/L), but it is not valid for RLS patients with normal serum ferritin. To the best of our knowledge, this NMA provided the most comprehensive review for the efficacy of currently available drugs in treatment of RLS, and may assist clinical decision. Additionally, we also analyzed the risk of common adverse effect of these 10 drugs.

Levodopa was first reported effective in RLS patients by Akpinar in 1987 (Akpinar, 1987). Several controlled studies subsequently proved its efficacy in idiopathic RLS (Benes et al., 1999; Saletu et al., 2003). Evidence-based guidelines ever had recommended levodopa as an effective therapy for RLS in some European countries (Littner et al., 2004). Unexpectedly, our NMA found levodopa is not effective in relieving RLS symptoms when compared with placebo. The inefficiency of levodopa in relieving RLS symptoms may be due to that, even though levodopa is well-proven to improve symptoms of RLS, it leads to later augmentation, which means that RLS symptoms in patients receiving levodopa will appear earlier than usual, be prolonged (even to daytime), get severer, and extend to body parts which are formerly unaffected (Garcia-Borreguero et al., 2015). It is reported that within 6 months of follow-up, 71% patients taking the medicine have suffered from the augmentation (Garcia-Borreguero et al., 2015). Hardly all of our included participants treated by these drugs for at least 12 weeks. We speculate that the augmentation of levodopa counteracts its effect on RLS. Moreover, the limited number of included studies may also lead to controversial pooled result. According to the numerous studies about its effectiveness in short time use and augmentation for long-term follow-up, a short-term treatment with intermittent levodopa administration is supposed to be a good choice (Silber et al., 2004). However, the optimal dosage, frequency and duration of levodopa treatment need further investigation. Apart from augmentation, nausea, vomiting, headache, dizziness, and fatigue are the classical side effects reported in levodopa use, most adverse events are mild to moderate in severity and well-tolerated (Garcia-Borreguero et al., 2015). In this NMA, we did not identify significantly increased incidence of adverse effects in patients receiving levodopa.

DAs have replaced levodopa as the first-line treatment for RLS, with support of several positive results of large multi-center trials (Earley, 2003; Hening et al., 2004; Vignatelli et al., 2006). Cabergoline is an ergot-derived dopamine agonist which shows mainly an agonistic activity at dopamine D2 receptors. It has the longest half-life (65 hours) among all DAs currently approved in humans, resulting in continuously and smoothly working. A large-scale controlled trial proved that cabergoline was superior to levodopa in relieving symptoms of RLS (Winkelmann et al., 2018). In our NMA, cabergoline showed the greatest efficacy in relieving RLS symptoms. However, the possible adverse effects of ergot-derived DAs, such as pericardial, retroperitoneal, and pleuropulmonary fibrosis, may limit the use of cabergoline, especially considering that pergolide, another ergot-derived DA, was alerted by US-FDA (U.S. Food and Drug Administration, 2003) (http://www.fda.gov/medwatch/SAFETY/). Though no direct evidence supports cabergoline will cause the same adverse effect as pergolide, use of cabergoline in treating RLS is not common, mostly due to concerns on safety. European RLS guidelines also no longer recommend cabergoline for treatment of RLS (García-Borreguero et al., 2012a). It is only suggested as a third option when other recommended drugs do not work according to the guidelines of USA (Aurora et al., 2012). However, in Benes's study, cabergoline is a safe and well-tolerated option for alleviating symptoms of idiopathic RLS within its 28-week follow-up (Benes et al., 2004). Generally, cabergoline may be a choice for RLS treatment only if its safety is well-confirmed.

Non-ergot derived DAs such as pramipexole, ropinirole, rotigotine are also widely used in patients with severe RLS due to their long half-lives (de Biase et al., 2014). In our NMA, most participants in the included studies were diagnosed as moderate or severe RLS (with a baseline total score of at least 15 points on the IRLS and a history of at least 15 nights of RLS symptoms during the previous month), ropinirole showed favorable efficacy in both primary and secondary RLS patients. Most open-label clinical trials have also reported that ropinirole significantly relieved RLS symptoms (Fulda and Wetter, 2005). In spite less augmentation was reported in patients treated with ropinirole (3.5%) compared to levodopa, 50% of these patients with augmentation discontinued ropinirole use (García-Borreguero et al., 2012b; Iftikhar et al., 2017). Pramipexole has a high selectivity for D2 and D3 receptors with a half-life of 8 to 12 hours. It is the second agent approved by FDA for the treatment of moderate-severe primary RLS patients (de Biase et al., 2014). Pramipexole is also beneficial for the comorbid symptoms such as depression, anxiety, sleep disorders which are common in RLS patients (Saletu et al., 2002). In our NMA, we found that pramipexole more effective in relieving RLS symptoms compared with ropinirole, which is consistent with previous studies (Kruszewski and Shane, 2007; Allen et al., 2014). However, it is noteworthy that 8–56% rates of augmentation was reported in RLS patients treated with pramipexole, even persisted for up to 10 years (Ferini-Strambi, 2002; Silver et al., 2011). In our NMA, we also found rotigotine effective in relieving RLS symptoms (García-Borreguero et al., 2012a; de Biase et al., 2014). Considering rotigotine providing a transdermal, continuous delivery, it is suitable for patients with daytime symptoms, swallowing difficulties, and undergoing surgery (Serafini et al., 2010). Importantly, similar to short-term studies in our NMA, rotigotine is also proven effective in patients with moderate-severe RLS for long-term administration (Serafini et al., 2010). Nausea, headache, somnolence, dizziness and orthostatic hypotension are the main adverse effects of non-ergot derived DAs. Among all analyzed drugs in our NMA, non-ergot derived DAs are most likely to induce nausea. Additionally, patients receiving pramipexole were reported likely to suffer from nasopharyngitis (Partinen et al., 2006), but our NMA did not report significant difference in the incidence of nasopharyngitis when comparing drugs to placebo, including pramipexole. Moreover, application site reactions and inconsistent absorption of rotigotine transdermal patch limit its clinic use (Braun et al., 2009; Oertel et al., 2011).

α-2-δ ligands, including gabapentin enacarbil, gabapentin and pregabalin have been widely used in RLS patients. Similarly, in our NMA, we found gabapentin effective in treatment of RLS, it has also been proven more effective in improving sleep and awakening quality compared with ropinirole (Saletu et al., 2010). Many randomized controlled trials have demonstrated the efficacy of gabapentin enacarbil in the treatment of RLS, it can significantly improve sleep quality, mood disturbances and quality of life vs. placebo in RLS (Ahmed et al., 2016; Avidan et al., 2016). Gabapentin enacarbil was designed to provide sustained gabapentin exposure and a delayed peak plasma concentration compared with an equivalent dose of gabapentin, resulting in overcome the pharmacokinetic deficiencies of gabapentin (Cundy et al., 2004, 2008). Theoretically, gabapentin enacarbil ought to have better efficacy in alleviating RLS symptoms than gabapentin for its pharmacokinetic character. However, our SUCRA showed an opposite tend. We attribute this result to the influence of limited participants and indirect comparison, because there is only one study on the efficacy of gabapentin in secondary RLS patients, and we could only compare gabapentin and gabapentin enacarbil indirectly due to lack of direct comparison. More direct comparison between gabapentin and gabapentin enacarbil is needed for further investigation. Furthermore, when studies on cabergoline are excluded, gabapentin is not effective to relieve symptoms of RLS in the forest plot, while SUCRA indicates gabapentin is a good choice for RLS patients just inferior to oxycodone-naloxone. We attribute this result to the following two reason: (1) SUCRA is calculated based on the results of NMA, and reflect the estimated probability of rank to each drugs instead of a specific value. That is, the SUCRA value represents the probability that a drug has among the best options. So SUCRA can sometimes mismatch with the clinical effect. (2) only one study researched the effect of gabapentin on secondary RLS patient is included in this NMA, the limited number and different participants may influence the pooled value calculated by the direct and indirect evidence (Rouse et al., 2017; Xu et al., 2018). Pregabalin has been proven effective for RLS, particularly in patients with comorbid anxiety, insomnia or neuropathic pain (de Biase et al., 2019). In our NMA, patients with moderate to severe RLS are also proven to benefit from pregabalin, which is consistent with previous studies (Allen et al., 2010). 300 mg/day of pregabalin could alleviate RLS symptoms more significantly than 0.5 mg/day of pramipexole while with less augmentation (Garcia-Borreguero et al., 2014). The most common adverse effects reported in α-2-δ ligands are dizziness and somnolence. Our NMA indicates significantly increasing risk of somnolence in pregabalin and gabapentin enacarbil use. α-2-δ ligands also tend to exacerbate risk of falls, cognitive impairment, depression and obesity/metabolic syndrome for long-term use with limited investigation (Garcia-Borreguero et al., 2016), but we did not analyze these adverse effects in our NMA due to lack of data. European RLS Study Group and the RLS Foundation has recommended α-2-δ ligands as first-line agents in treating RLS for their high efficacy, low augmentation risk and well-tolerated (Garcia-Borreguero et al., 2016). However, only Gabapentin enacarbil in α-2-δ ligands is approved by the US FDA currently.

Iron supplement was proven to be effective in RLS patients with low serum ferritin level (Anguelova et al., 2020). Whether it would be benefit for patients with normal ferritin levels remains unclear. Our study confirms the efficacy of iron in decreasing IRLS scores in RLS patient with serum ferritin lower than 45 ug/L, however, the effectiveness of iron supplement could not be proven in RLS patients with serum ferritin upper than 45 ug/L. Previous guidelines also supported the used of both oral and intravenous iron supplement in treatment of RLS patients with lower serum ferritin level (Winkelman et al., 2016; Allen et al., 2018). Moreover, given that brain iron insufficiency is underlying pathology of RLS (Earley et al., 2014), it should be noted that the aim of iron supplement for RLS patient is to increase the level of brain iron through increasing the level of peripheral iron store. Therefore, brain iron store, rather than peripheral iron store, should be considered when clinicians decide to use iron supplement for the treatment of RLS. Iron supplement is not commonly used in primary RLS in clinic despite it tends to be effective. To further investigate the benefits and limitations of iron supplement, more researches with wide serum ferritin range and brain iron level are needed for RLS patients.

Opioids have been found effective in the treatment of RLS, but they are usually used in patients failed in dopaminergic drug treatment (Walters et al., 1993; Ondo, 2005). There is only one study (Trenkwalder et al., 2013) indicates that prolonged release oxycodone-naloxone significantly relieved symptoms of RLS in patients who failed in previous treatments (mostly dopaminergic). It should be noticed that opioids could cause addiction and abuse in patients and worsen sleep apnea. Therefore, prolonged release oxycodone-naloxone is assessed only for second-line treatment in severe RLS patients. In our NMA, the SUCRA indicates that ropinirole exhibits similar efficacy in relieving RLS symptoms compared to prolonged release oxycodone-naloxone with both participants are moderate to severe and severe-very severe RLS patients. We speculate that, ropinirole may be a promising choice in severe or very severe RLS patients if further researched, especially considering it does not cause addition. A review summarized studies published in PubMed between 1993 and 2016 concluded that opioids could be used as additional therapy to either or both dopamine agonists and α-2-δ ligands (combination therapy), or can be used alone as monotherapy (de Biase et al., 2016). Garcia-Borreguero' review also suggested a low dose of an opioid (prolonged-release oxycodone or methadone) use in patients with severe augmentation instead of α-2-δ ligands (Garcia-Borreguero et al., 2016). When patients are selected appropriately, low-dose opioid therapy is typically very effective and safe even when used for long-term therapy (Garcia-Borreguero et al., 2016).

To further investigate the efficacy of different drugs in primary RLS, we conducted a separate analysis based on patients diagnosed with primary RLS. For patients with primary RLS, we found both α-2-δ ligands and DAs are effective. As a previous study mentioned, for patents with primary RLS, ropinirole, gabapentin enacarbil and pregabalin were recommended to relieve subjective sleep parameters (such as total sleep time sleep latency and sleep efficiency), (Brindani et al., 2009; Winkelman et al., 2016). Given the risks of cardiac valvulopathy of cabergoline, we still recommended non-ergot-derived DAs (pramipexole, ropinirole, rotigotine) and α-2-δ ligands as appropriate options for primary RLS. For patients with secondary RLS, there is insufficient evidence to support the use of drug therapy in this NMA. Considering complicated factors affect severity and symptoms of RLS, separate investigations of treatments for secondary RLS caused by different factors is necessary. As reported, etiological treatment of secondary RLS may result in improvement or resolution of symptoms (Bayard et al., 2008).

There are several limitations should be noticed. First, IRLS can only generally reflect efficacy of different drugs, it is unable to reflect changes of life quality and improvement of the periodic limb movements during sleep particularly, considering RLS is a sleep-related disorder and related to quality of patients' lives, IRLS may not reflect RLS as a whole, but only reflect the symptoms. Second, we did not analyze the efficacy of different drugs in patients with different severity. Most participants included in our NMA had severe RLS. Third, some analyzed drugs were taken by limited number of patients, which may lead to bias in our NMA. More randomized studies of large sample size are needed to further investigate the efficacy of oxycodone-naloxone. Forth, due to the short duration of the included studies and lack of data, we only analyzed some common adverse effect and did not evaluate the optimal dosage of each drug. More detailed and longer-term studies on adverse effects are needed. Fifth, due to insufficient data, we did not analyze the augmentation of dopaminergic drugs and DAs. Clarifying the onset time of augmentation and dosage of dopaminergic drugs may be meaningful.

Our NMA used currently available evidences to comprehensively compare the efficacy and safety of various drugs widely used in clinic for RLS. Direct and indirect evidence is pooled together to provide updated evidences. Cabergoline showed the greatest efficacy in relieving symptoms of RLS, while its use is limited for possible side effect of fibrosis. α-2-δ ligands and DAs are favorable choices for their significant efficacy and good tolerability in both primary and secondary RLS. α-2-δ ligands prefer to be selected for less augmentation than DAs according to the previous investigations. In addition, iron supplement can significantly alleviate symptoms of RLS patients with iron deficiency than placebo. Oxycodone-naloxone could be considered in patients with severe or very severe RLS who failed in treatment with above drugs. Further researches with a large number of participants are needed for a better selection of these drugs.

## Data Availability Statement

The original contributions presented in the study are included in the article/Supplementary Material, further inquiries can be directed to the corresponding authors.

## Author Contributions

XZ, LM, and LC: conception and design. XZ, JD, and LM: acquisition of data. XZ, CT, and LM: analysis and interpretation of data. XZ and LM: drafting the article. XL, CT, and LC: critically revising the article. XZ, JD, YL, CD, LZ, XL, CT, LM, and LC: reviewed submitted version of manuscript. LC: approved the final version of the manuscript on behalf of all authors. XZ, JD, and LM: statistical analysis. XL, CT, and LC: administrative/technical/material support. LC: supervision. All authors contributed to the article and approved the submitted version.

## Funding

This work was funded by National Natural Science Foundation of China (81771391 and 82001367), Kuanren talent program of the Second Affiliated Hospital of Chongqing Medical University.

## Conflict of Interest

The authors declare that the research was conducted in the absence of any commercial or financial relationships that could be construed as a potential conflict of interest.

## Publisher's Note

All claims expressed in this article are solely those of the authors and do not necessarily represent those of their affiliated organizations, or those of the publisher, the editors and the reviewers. Any product that may be evaluated in this article, or claim that may be made by its manufacturer, is not guaranteed or endorsed by the publisher.
