# Supplementary material for: The Efficacy and Safety of Pharmacological Treatments for Restless Legs Syndrome: Systemic Review and Network Meta-Analysis
Source: Front Neurosci. 2021 Oct 26;15:751643. doi: 10.3389/fnins.2021.751643 (PMC8576256; doi:10.3389/fnins.2021.751643)
Supplement: Supplementary file 1 [file Data_Sheet_1.pdf]

Supplementary Material

1 Supplementary Figures and Tables

1.1 Supplementary Figures

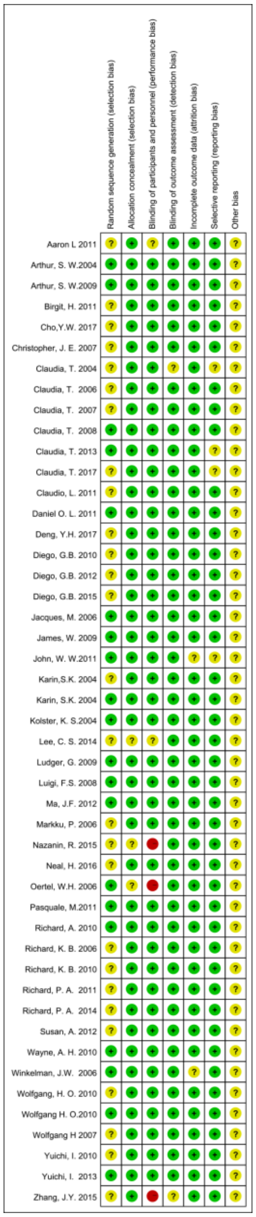

Supplementary Figure 1. Risk bias of randomized controlled studies

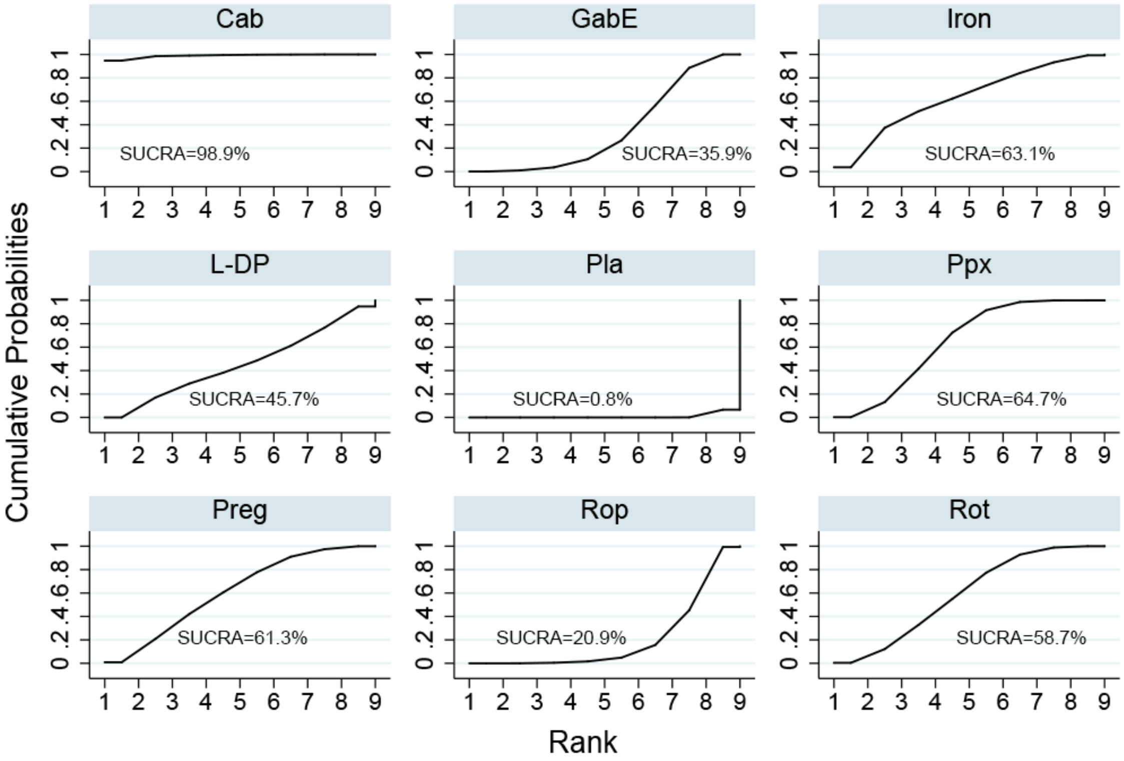

Supplementary Figure 2. Surface Under the Cumulative Ranking Curve of treatments for primary RLS patients.

1.2 Supplementary Tables

Supplementary Table 1. The characteristic of included studies

Source	Country	Diagnosis	Type of interventions	Sample size	Gender (Female%)	Age(year)	Duration(year)	change of IRLS scores from baseline(mean±SD)
Karin, S.K. 2004	Germany	primary RLS	Rotigotine	49	73.7	58.3± 8.7	10.3±10.9	-15.7±8.28
			Placebo	14	50	60.1±8.5	8.4 ± 7.0	-7.4±8.23
Wolfgang, H. 2007	Austria, Germany, et al.	primary RLS	Pramipexole	224	64.3	55.4±11.6	4.95±9.2	-12.3±8.98
			Placebo	114	68.4	55.8±10.9	5.63±9.1	-5.7±9.61
Christopher, J. E. 2007	USA	primary RLS	Iron	11	55	66.4±11.4	NA	-10.1±5.10
			Placebo	7	71	61.4±10.0	NA	-12.0±11.50
Claudia, T. 2007	NA	primary RLS	Cabergoline	178	67.4	56.9±11.7	11.0±12.0	-16.1±10.20
			Levodopa	183	74.9	58.7±11.6	12.1± 13.6	-9.6±9.70
Richard, K. B. 2010	USA	primary RLS	Gabapentin Enacarbil	96	65	50.7±11.68	12.3±12.7	-3.2±6.11
			Placebo	97	54	52.2±12.13	15.7±15.3	-1.4±5.79
Nazanin, R. 2015	Iran	secondary RLS	Gabapentin	42	42.9	57.8±13.7	NA	-17.4±5.24
			Levodopa	40	45	52.6±11.5	NA	-13.4±6.61
Susan, A. 2012	USA	primary RLS	Gabapentin Enacarbil	508	60	48.1±12.71	13.5±12.82	-13.6±9.00
			Placebo	244	62	49.3±12.32	13.9±12.99	-9.3±8.59
James, W. 2009	USA	primary RLS	Iron	11	54.5	mean age 60	NA	-10.3±7.40
			Placebo	7	71.4	mean age 58	NA	-1.1±5.60
Richard, K. B. 2006	USA	primary RLS	Ropinirole	187	58.3	52.2±12.79	NA	-13.6±6.47
			Placebo	193	63.7	52.4±13.15	NA	-9.7±7.97
Ma, J.F. 2012	China	primary RLS	Pramipexole	203	60.4	56.46±11.88	NA	-15.8±10.31
			Placebo	103	72.8	56.86±11.89	NA	-11.4±9.33
Arthur, S. W.2004	USA	primary RLS	Ropinirole	131	58	54.9±10.87	NA	-11.2±8.69
			Placebo	135	61.5	56.0±11.25	NA	-8.7±8.71

Luigi, F.S. 2008	USA	NA	Pramipexole	182	72.5	56.3±12.4	5.36±9.8	-13.4±9.44
			Placebo	187	63.6	56.9±13.0	5.66±9.9	-9.6±9.57
Yuichi, I. 2010	Japan	primary RLS	Pramipexole	20	55	48.7±16.1	0.2±0.6	-16.1±7.39
			Placebo	21	47.6	62.3±11.9	0.6 ± 1.3	-6.4±7.98
Claudia, T. 2008	Austria, Finland, et al.	primary RLS	Rotigotine	333	73	57.3±12.1	NA	-16.8±9.52
			Placebo	114	70	59.7±10.0	NA	-8.6±9.60
Ludger, G. 2009	Sweden	primary RLS	Iron	29	86.2	47.0 ±10.0	NA	-8.7±9.40
			Placebo	31	90.3	46.0±8.0	NA	-6.9±9.70
Wayne, A. H. 2010	USA	primary RLS	Rotigotine	395	63	53.2±12.2	2.5±4.7	-14.3±9.4
			Placebo	99	57	52.8±12.6	2.1±4.4	-9.0±7.70
Claudia, T. 2013	Austria, Germany, et al.	NA	Oxycodone-naloxone	132	65	NA	10.2±9.8	-19.3±8.23
			Placebo	144	68	NA	10.4±10.2	-13.6±10.75
Kolster, K. S.2004	Germany	NA	Cabergoline	62	50	56.4±9.1	18.1±12.6	-15.7±11.9
			Placebo	22	81.8	55.6±9.2	19.8±14.2	-3.3±8.00
Markku, P. 2006	Finland	primary RLS	Pramipexole	83	81.8	60.0±10.1	5.1±11.1	-17.0±6.94
			Placebo	21	81	53.3±11.1	2.7±10.1	-6.1±6.92
Deng, Y.H. 2017	China	secondary RLS	Iron	16	NA	63.63±4.83	NA	-7.4±2.03
			Placebo	16	NA	64.19±7.93	NA	-0.8±2.61
Pasquale, M.2011	Finland, France, et al.	primary RLS	Pramipexole	203	67	55.0±13.8	3.5±7.2	-14.2±9.97
			Placebo	200	73	56.1±12.1	3.3 ±6.5	-8.1±9.89
John, W. W.2011	USA	primary RLS	Gabapentin Enacarbil	131	NA	52.0±12.7	NA	-15.0±8.30
			Placebo	131	NA	52.0±12.7	NA	-8.4±8.16
Oertel, W.H. 2006	USA	primary RLS	Cabergoline	20	70	57.3±10.3	13.6 ±10.3	-23.7±11.20
			Placebo	20	75	55.5±9.3	22.1 ±16.0	-7.9±11.00
Claudia, T. 2017	Germany	NA	Iron	59	81.4	53.0±15.7	NA	-9.6±10.7
			Placebo	51	82.4	55.5±15.9	NA	-5.0±10.71
Birgit, H. 2011	European countries	primary RLS	Pramipexole	166	61.4	57.9±12.7	6.0±9.6	-13.7±10.31
			Placebo	163	57.7	55.8±11.4	5.4±8.5	-11.1±10.21
Wolfgang, H. O. 2010	Austria, Finland, et al.	primary RLS	Rotigotine	46	76	60.8±9.4	1.8±2.9	-16.5±9.30
			Placebo	20	70	56.3±9.8	3.1±8.0	-9.9±9.90

Richard, A. 2010	USA	primary RLS	Pregabalin	114	59.1	50.5±11.9	mean 7.5	-16.0±8.91
			Placebo	23	56.5	50.3±10.5	mean 8.7	-7.7±8.15
Wolfgang, H. O. 2010	NA	primary RLS	Rotigotine	280	68.6	58.9±9.9	NA	-17.3±10.5
			Placebo	53	60.4	58.5±11.4	NA	-9.3±9.60
Zhang, J.Y. 2015	China	primary RLS	Pramipexole	102	65.7	53.6±12.9	1.62±0.7	-13.2±7.07
			Placebo	102	61.8	52.9±13.1	1.59±0.6	-9.4±6.06
Richard, P. A. 2014	USA	primary RLS	Pregabalin	182	67.6	54.3±13.0	NA	-11.4±6.65
			Pramipexole	358	55	54.2±13.5	NA	-10.1±6.65
			Placebo	179	62	53.5±13.3	NA	-6.9±6.48
Winkelman, J.W. 2006	USA	primary RLS	Pramipexole	254	66.7	53.4±12.7	4.9 ±15.0	-14.0±9.33
			Placebo	85	63.5	51.5±14.0	5.2±15.0	-9.3±9.22
Richard, P. A. 2011	USA	NA	Iron	24	70.1	49.5±11.4	NA	-8.9±8.50
			Placebo	19	52.6	54.8±13.6	NA	-4.0±6.10
Diego, G.B. 2010	Spain	primary RLS	Pregabalin	30	NA	48.23±13.2	13.63±10.3	-13.0±5.99
			Placebo	28	NA	53.0±14.7	16.79±13.2	-10.3±7.46
Lee, C. S. 2014	Korea	NA	Iron	15	93.3	53.3±13.0	NA	-9.1±7.07
			Pramipexole	15	100	59.1±10.83	NA	-8.7±8.31
Aaron, L. 2011	USA	primary RLS	Gabapentin Enacarbil	197	60.4	49.5±12.0	NA	-15.4±8.96
			Placebo	376	57.7	50.6±11.8	NA	-14.8±8.64
Yuichi, I. 2013	Japan	primary RLS	Rotigotine	189	48.9	50.7±13.3	13.4±13.1	-14.6±9.00
			Placebo	95	56.8	53.4±15.3	15.7±14.4	-14.3±8.9
Arthur, S.W. 2009	NA	primary RLS	Gabapentin Enacarbil	62	67	51.9±11.2	NA	-16.1±7.93
			Placebo	33	52	49.4±10.9	NA	-8.9±7.72
Claudio, L. 2011	Switzerland	primary RLS	Pramipexole	31	NA	57.0±11 .0	NA	-7.2±9.50
			Levodopa	28	NA	57.0±11 .0	NA	-4.0±7.50
Neal, H. 2016	USA	NA	Gabapentin enacarbil	233	58	48.1±12.7	13.5 ±12.8	-14.9±9.59
			Placebo	133	62	49.3±12.3	13.9 ±12.9	-9.5±8.62
Diego, G.B. 2015	USA	primary RLS	Rotigotine	101	57.4	47.9±14.2	mean2.3	-14.9±9.30
			Placebo	49	40.8	47.9±13.7	mean 2.0	-12.7±7.60
Claudia, T. 2006	Germany	primary RLS	Ropinirole	45	47.6	54.0±11.13	NA	-15.5±6.74
			Placebo	47	63.8	56.2±11.2	NA	-14.8±6.63
Diego, G.B. 2012	NA	primary RLS	Ropinirole	196	63	56.5±11.9	NA	-15.9±10.64
			Placebo	205	63	56.1±11.4	NA	-13.4±11.02

Cho, Y.W. 2017	Korea	primary RLS	Iron	32	81.3	47.3±13.3	12.9±14.3	-8.3±7.50
			Placebo	32	81.3	51.5±12.0	9.0±8.8	-4.8±8.70
Daniel, O.L. 2011	USA	primary RLS	Gabapentin Enacarbil	207	59	48.3±12.8	13.5 ±13.1	-13.0±7.08
			Placebo	96	59	49.1±12.2	14.4 ±12.9	-9.8±6.85
Claudia, T. 2004	European countries	NA	Ropinirole	146	60.3	54.0±11.1	16.4±14.1	-11.4±11.72
			Placebo	138	65.9	56.2±11.2	17.3±14.9	-8.03±8.67
Jacques, M. 2004	Australia, Austria, et al.	primary RLS	Ropinirole	131	46.7	51.6±11.33	22.0±15.31	-11.2±8.69
			Placebo	135	63.8	55.3±11.03	23.6±15.35	-8.7±8.71

MD, mean difference; NA, not applicable; RLS, Restless leg syndrome; SD, standard deviation

Supplementary Table 2. Network meta-analysis result of common side effects of pharmacological treatments for RLS

	Levodopa	Placebo	Gabapentin enacarbil	Pregabalin	Oxycodone–naloxone	Cabergoline	Rotigotine	Pramipexole	Ropinirole	Iron
(a)Nausea as a side effect [OR (95% CI)]										
Levodopa		1.79 (0.23,13.93)	1.81 (0.21,15.43)	3.52 (0.27,45.32)	3.75 (0.30,47.06)	3.86 (0.93,16.05)	4.48 (0.52,38.77)	5.71 (0.65,50.51)	8.98 (1.03,78.30)	16.11 (0.32,823.27)
Placebo	0.56 (0.07,4.35)		1.01 (0.55,1.87)	1.96 (0.43,9.04)	2.10 (0.48,9.19)	2.16 (0.49,9.44)	2.50 (1.28,4.87)	3.19 (1.53,6.67)	5.02 (2.50,10.06)	9.00 (0.31,258.16)
Gabapentin enacarbil	0.55 (0.06,4.70)	0.99 (0.53,1.83)		1.94 (0.37,10.05)	2.07 (0.42,10.26)	2.13 (0.43,10.53)	2.47 (1.00,6.12)	3.15 (1.21,8.22)	4.95 (1.96,12.53)	8.89 (0.29,269.57)
Pregabalin	0.28 (0.02,3.66)	0.51 (0.11,2.34)	0.52 (0.10,2.67)		1.07 (0.13,8.93)	1.10 (0.13,9.16)	1.27 (0.24,6.74)	1.62 (0.40,6.59)	2.55 (0.48,13.65)	4.58 (0.11,182.89)
Oxycodone–naloxone	0.27 (0.02,3.34)	0.48 (0.11,2.09)	0.48 (0.10,2.39)	0.94 (0.11,7.85)		1.03 (0.13,8.31)	1.19 (0.24,6.04)	1.52 (0.29,7.94)	2.39 (0.47,12.26)	4.29 (0.11,168.04)
Cabergoline	0.26 (0.06,1.08)	0.46 (0.11,2.03)	0.47 (0.09,2.32)	0.91 (0.11,7.61)	0.97 (0.12,7.85)		1.16 (0.23,5.87)	1.48 (0.28,7.70)	2.33 (0.46,11.88)	4.17 (0.11,163.29)
Rotigotine	0.22 (0.03,1.94)	0.40 (0.21,0.78)	0.40 (0.16,1.00)	0.79 (0.15,4.16)	0.84 (0.17,4.24)	0.86 (0.17,4.37)		1.28 (0.47,3.45)	2.01 (0.76,5.27)	3.60 (0.12,110.25)
Pramipexole	0.17 (0.02,1.55)	0.31 (0.15,0.65)	0.32 (0.12,0.83)	0.62 (0.15,2.50)	0.66 (0.13,3.42)	0.68 (0.13,3.51)	0.78 (0.29,2.12)		1.57 (0.57,4.32)	2.82 (0.09,87.57)
Ropinirole	0.11 (0.01,0.97)	0.20 (0.10,0.40)	0.20 (0.08,0.51)	0.39 (0.07,2.09)	0.42 (0.08,2.14)	0.43 (0.08,2.20)	0.50 (0.19,1.31)	0.64 (0.23,1.75)		1.79 (0.06,55.27)
Iron	0.06 (0.00,3.17)	0.11 (0.00,3.19)	0.11 (0.00,3.41)	0.22 (0.01,8.72)	0.23 (0.01,9.12)	0.24 (0.01,9.37)	0.28 (0.01,8.51)	0.35 (0.01,11.02)	0.56 (0.02,17.17)	
(b)Fatigue as a side effect [OR (95% CI)]										
Levodopa		1.56 (0.25,9.74)	1.57 (0.24,10.16)	2.65 (0.36,19.25)	4.32 (0.63,29.82)	3.08 (1.32,7.21)	1.74 (0.26,11.65)	2.09 (0.32,13.67)	4.26 (0.63,29.03)	0.29 (0.01,13.29)
Placebo	0.64 (0.10,4.03)		1.01 (0.71,1.43)	1.70 (0.80,3.62)	2.78 (1.52,5.08)	1.98 (0.39,10.09)	1.12 (0.68,1.83)	1.34 (0.89,2.02)	2.74 (1.56,4.81)	0.19 (0.01,5.34)
Gabapentin enacarbil	0.64 (0.10,4.12)	0.99 (0.70,1.40)		1.69 (0.74,3.87)	2.75 (1.37,5.51)	1.96 (0.37,10.35)	1.11 (0.61,2.02)	1.33 (0.78,2.27)	2.71 (1.40,5.26)	0.19 (0.01,5.38)
Pregabalin	0.38 (0.05,2.74)	0.59 (0.28,1.25)	0.59 (0.26,1.36)		2.07 (1.00,4.29)	1.16 (0.19,6.99)	0.66 (0.27,1.62)	0.79 (0.41,1.50)	1.61 (0.63,4.10)	0.11 (0.00,3.41)
Oxycodone–naloxone	0.23 (0.03,1.59)	0.36 (0.20,0.66)	0.36 (0.18,0.73)	0.61 (0.23,1.61)		0.71 (0.13,4.04)	0.40 (0.19,0.88)	0.48 (0.23,1.00)	0.99 (0.43,2.25)	0.07 (0.00,2.03)
Cabergoline	0.32 (0.14,0.76)	0.50 (0.10,2.56)	0.51 (0.10,2.69)	0.86 (0.14,5.16)	1.40 (0.25,7.94)		0.40 (0.19,0.88)	0.68 (0.13,3.62)	1.38 (0.25,7.72)	0.09 (0.00,3.92)
Rotigotine	0.17 (0.01,4.94)	0.89 (0.55,1.46)	0.90 (0.49,1.65)	1.52 (0.62,3.74)	2.48 (1.14,5.40)	1.77 (0.32,9.68)		1.20 (0.63,2.27)	2.45 (1.16,5.17)	0.17 (0.01,4.94)
Pramipexole	0.14 (0.00,4.08)	0.75 (0.50,1.12)	0.75 (0.44,1.29)	1.27 (0.67,2.42)	2.07 (1.00,4.29)	1.48 (0.28,7.91)	0.84 (0.44,1.58)		2.04 (1.02,4.09)	0.14 (0.00,4.08)
Ropinirole	0.23 (0.03,1.60)	0.36 (0.21,0.64)	0.37 (0.19,0.71)	0.62 (0.24,1.59)	1.01 (0.45,2.31)	0.72 (0.13,4.04)	0.41 (0.19,0.86)	0.49 (0.24,0.98)		0.07 (0.00,2.04)
Iron	3.41 (0.08,154.84)	5.31 (0.19,150.49)	5.36 (0.19,154.83)	9.05 (0.29,278.85)	14.76 (0.49,441.64)	10.53 (0.26,434.16)	5.95 (0.20,174.88)	7.12 (0.25,207.07)	14.55 (0.49,432.35)	
(c)Headache as a side effect [OR (95% CI)]										
Levodopa		3.21 (0.22,47.88)	3.61 (0.22,58.04)	2.18 (0.11,42.73)	6.43 (0.26,161.32)	1.52 (0.28,8.42)	2.59 (0.16,43.23)	3.61 (0.22,59.55)	3.62 (0.22,60.32)	2.77 (0.07,117.14)
Placebo	0.31 (0.02,4.64)		1.12 (0.59,2.15)	0.68 (0.19,2.36)	2.00 (0.34,11.60)	0.47 (0.06,3.83)	0.81 (0.37,1.77)	1.12 (0.53,2.37)	1.13 (0.51,2.47)	0.86 (0.06,11.52)
Gabapentin enacarbil	0.28 (0.02,4.47)	0.89 (0.47,1.71)		0.60 (0.15,2.46)	1.78 (0.27,11.61)	0.42 (0.05,3.77)	0.72 (0.26,1.99)	1.00 (0.37,2.69)	1.00 (0.36,2.78)	0.77 (0.05,11.12)
Pregabalin	0.46 (0.02,9.00)	1.47 (0.42,5.14)	1.65 (0.41,6.73)		2.95 (0.34,25.46)	0.70 (0.06,7.98)	1.19 (0.27,5.16)	1.66 (0.48,5.78)	1.66 (0.38,7.23)	1.27 (0.07,22.58)
Oxycodone–naloxone	0.16 (0.01,3.91)	0.50 (0.09,2.90)	0.56 (0.09,3.66)	0.34 (0.04,2.93)		0.24 (0.02,3.64)	0.40 (0.06,2.77)	0.56 (0.08,3.79)	0.56 (0.08,3.86)	0.43 (0.02,9.88)
Cabergoline	0.66 (0.12,3.64)	2.11 (0.26,17.09)	2.37 (0.27,21.17)	1.43 (0.13,16.35)	4.22 (0.27,64.86)		1.70 (0.18,15.91)	2.37 (0.26,21.86)	2.38 (0.26,22.20)	1.82 (0.07,50.89)
Rotigotine	0.39 (0.02,6.43)	1.24 (0.56,2.72)	1.39 (0.50,3.85)	0.84 (0.19,3.64)	2.48 (0.36,17.00)	0.59 (0.06,5.48)		1.39 (0.47,4.11)	1.40 (0.46,4.23)	1.07 (0.07,16.04)
Pramipexole	0.28 (0.02,4.56)	0.89 (0.42,1.87)	1.00 (0.37,2.68)	0.60 (0.17,2.10)	1.78 (0.26,12.00)	0.42 (0.05,3.88)	0.72 (0.24,2.12)		1.00 (0.34,2.95)	0.77 (0.05,11.37)
Ropinirole	0.28 (0.02,4.60)	0.89 (0.41,1.94)	1.00 (0.36,2.75)	0.60 (0.14,2.62)	1.77 (0.26,12.16)	0.42 (0.05,3.92)	0.72 (0.24,2.17)	1.00 (0.34,2.94)		0.77 (0.05,11.47)
Iron	0.36 (0.01,15.22)	1.16 (0.09,15.44)	1.30 (0.09,18.78)	0.79 (0.04,13.93)	2.32 (0.10,52.99)	0.55 (0.02,15.31)	0.93 (0.06,14.01)	1.30 (0.09,19.28)	1.31 (0.09,19.54)	

(d)Dizziness as a side effect [OR (95% CI)]

Levodopa		5.44 (0.51,58.28)	20.11 (1.75,230.97)	15.44 (0.96,248.78)	19.34 (1.04,359.53)	2.89 (0.68,12.30)	6.49 (0.53,80.26)	4.81 (0.36,64.05)	13.77 (1.10,172.80)	7.25 (0.17,308.11)
Placebo	0.18 (0.02,1.97)		3.70 (2.08,6.58)	2.84 (0.67,12.09)	3.56 (0.65,19.61)	0.53 (0.08,3.48)	1.19 (0.52,2.75)	0.88 (0.31,2.50)	2.53 (1.05,6.10)	1.33 (0.07,24.33)
Gabapentin enacarbil	0.05 (0.00,0.57)	0.27 (0.15,0.48)		0.77 (0.16,3.65)	0.96 (0.16,5.83)	0.14 (0.02,1.02)	0.32 (0.12,0.89)	0.24 (0.07,0.78)	0.68 (0.24,1.96)	0.36 (0.02,6.96)
Pregabalin	0.06 (0.00,1.04)	0.35 (0.08,1.50)	1.30 (0.27,6.18)		1.25 (0.13,11.75)	0.19 (0.02,2.00)	0.42 (0.08,2.23)	0.31 (0.09,1.09)	0.89 (0.16,4.85)	0.47 (0.02,12.05)
Oxycodone–naloxone	0.05 (0.00,0.96)	0.28 (0.05,1.55)	1.04 (0.17,6.30)	0.80 (0.09,7.49)		0.15 (0.01,1.89)	0.34 (0.05,2.24)	0.25 (0.03,1.83)	0.71 (0.10,4.85)	0.37 (0.01,10.88)
Cabergoline	0.35 (0.08,1.47)	1.88 (0.29,12.31)	6.96 (0.98,49.64)	5.35 (0.50,57.29)	6.70 (0.53,84.72)		2.25 (0.29,17.55)	1.67 (0.19,14.23)	4.77 (0.60,37.91)	2.51 (0.08,79.71)
Rotigotine	0.15 (0.01,1.90)	0.84 (0.36,1.93)	3.10 (1.13,8.51)	2.38 (0.45,12.63)	2.98 (0.45,19.91)	0.44 (0.06,3.47)		0.74 (0.20,2.80)	2.12 (0.63,7.12)	1.12 (0.05,22.90)
Pramipexole	0.21 (0.02,2.77)	1.13 (0.40,3.19)	4.18 (1.28,13.68)	3.21 (0.92,11.23)	4.02 (0.55,29.62)	0.60 (0.07,5.13)	1.35 (0.36,5.10)		2.86 (0.73,11.14)	1.51 (0.07,32.89)
Ropinirole	0.07 (0.01,0.91)	0.39 (0.16,0.95)	1.46 (0.51,4.18)	1.12 (0.21,6.11)	1.41 (0.21,9.58)	0.21 (0.03,1.67)	0.47 (0.14,1.58)	0.35 (0.09,1.36)		0.53 (0.03,10.94)
Iron	0.14 (0.00,5.86)	0.75 (0.04,13.68)	2.77 (0.14,53.57)	2.13 (0.08,54.68)	2.67 (0.09,77.48)	0.40 (0.01,12.66)	0.90 (0.04,18.38)	0.66 (0.03,14.49)	1.90 (0.09,39.47)	

(e)Somnolence as a side effect [OR (95% CI)]

Placebo		3.56 (1.77,7.14)	4.67 (1.23,17.66)	2.51 (0.38,16.73)	2.60 (0.08,80.47)	1.49 (0.59,3.77)	1.69 (0.51,5.61)	2.31 (0.63,8.39)	
Gabapentin enacarbil	0.28 (0.14,0.56)		1.31 (0.29,5.89)	0.70 (0.09,5.32)	0.73 (0.02,24.26)	0.42 (0.13,1.33)	0.48 (0.12,1.90)	0.65 (0.15,2.81)	
Pregabalin	0.21 (0.06,0.81)	0.76 (0.17,3.43)		0.54 (0.05,5.46)	0.56 (0.01,22.13)	0.32 (0.06,1.62)	0.36 (0.09,1.44)	0.49 (0.08,3.16)	
Oxycodone–naloxone	0.40 (0.06,2.66)	1.42 (0.19,10.71)	1.86 (0.18,18.90)		1.04 (0.02,52.38)	0.59 (0.07,4.91)	0.68 (0.07,6.37)	0.92 (0.09,9.13)	
Cabergoline	0.38 (0.01,11.87)	1.37 (0.04,45.31)	1.79 (0.05,71.08)	0.96 (0.02,48.59)		0.57 (0.02,19.99)	0.65 (0.02,24.65)	0.89 (0.02,34.64)	
Rotigotine	0.67 (0.27,1.70)	2.39 (0.75,7.62)	3.13 (0.62,15.89)	1.68 (0.20,13.93)	1.75 (0.05,61.17)		1.14 (0.25,5.18)	1.55 (0.32,7.60)	
Pramipexole	0.59 (0.18,1.95)	2.10 (0.53,8.39)	2.75 (0.69,10.94)	1.48 (0.16,13.96)	1.54 (0.04,58.18)	0.88 (0.19,4.00)		1.36 (0.23,7.92)	
Ropinirole	0.43 (0.12,1.58)	1.54 (0.36,6.69)	2.02 (0.32,12.92)	1.09 (0.11,10.79)	1.13 (0.03,44.12)	0.65 (0.13,3.16)	0.73 (0.13,4.27)		

(f)Nasopharyngitis as a side effect [OR (95% CI)]		0.93 (0.57,1.53)	0.95 (0.24,3.76)			1.45 (0.49,4.32)	1.03 (0.52,2.05)	1.23 (0.53,2.86)
Placebo	1.08 (0.65,1.77)		1.02 (0.24,4.42)			1.56 (0.47,5.18)	1.11 (0.47,2.59)	1.32 (0.49,3.52)
Gabapentin enacarbil	1.05 (0.27,4.19)	0.98 (0.23,4.25)				1.53 (0.27,8.86)	1.09 (0.33,3.59)	1.29 (0.26,6.53)
Pregabalin	0.69 (0.23,2.04)	0.64 (0.19,2.12)	0.65 (0.11,3.76)				0.71 (0.20,2.55)	0.84 (0.21,3.34)
Rotigotine	0.97 (0.49,1.93)	0.90 (0.39,2.11)	0.92 (0.28,3.04)			1.41 (0.39,5.10)		1.19 (0.40,3.55)
Pramipexole	0.81 (0.35,1.90)	0.76 (0.28,2.02)	0.77 (0.15,3.89)			1.18 (0.30,4.70)	0.84 (0.28,2.49)	

CI, confidence interval; OR, odds ratio; RLS, restless legs syndrome

Supplementary Table 3. Surface Under the Cumulative Ranking Curve (SUCRA) of all included drugs.

Treatment	Efficacy SUCRA	PrBest	MeanRank
Placebo	0.4	0.0	11.0
Gabapentin	80.1	10.1	3.0
Gabapentin enacarbil	32.6	0.0	7.7
Ropinirole	16.8	0.0	9.3
Rotigotine	54.7	0.1	5.5
Pramipexole	57.2	0.0	5.3
Iron	51.0	0.0	5.9
Levodopa	41.5	0.0	6.9
Pregabalin	56.4	0.2	5.4
Cabergoline	98.7	87.7	1.1
Oxycodone-naloxone	60.7	1.8	4.9

Supplementary Table 4. Surface Under the Cumulative Ranking Curve (SUCRA) of the rest of drugs after excluding cabergoline.

Treatment	Efficacy SUCRA	PrBest	MeanRank
Placebo	3.7	0.0	9.7
Gabapentin	69.5	40.2	3.7
Gabapentin enacarbil	42.6	0.2	6.2
Ropinirole	24.4	0.0	7.8
Rotigotine	66.6	8.3	4.0
Pramipexole	66.9	3.4	4.0
Iron	62.7	6.0	4.4
Levodopa	23.4	0.2	7.9
Pregabalin	68.7	12.8	3.8
Oxycodone-naloxone	71.6	28.8	3.6
